# Repository corticotropin injection versus corticosteroids for protection against renal damage in a focal segmental glomerulosclerosis rodent model

**DOI:** 10.1186/s12882-020-01879-6

**Published:** 2020-06-15

**Authors:** Kyle Hayes, Elizabeth Warner, Chris Bollinger, Dale Wright, Richard M. Fitch

**Affiliations:** Mallinckrodt Pharmaceuticals, 675 James S. McDonnell Blvd, 20-1-W, Hazelwood, MO USA

**Keywords:** Focal segmental glomerulosclerosis, Repository corticotropin injection, Nephrotic syndrome, Adrenocorticotropic hormone, Melanocortin receptor, Puromycin aminonucleoside

## Abstract

**Background:**

Focal segmental glomerulosclerosis (FSGS) causes renal fibrosis and may lead to kidney failure. FSGS and its common complication, proteinuria, are challenging to treat. Corticosteroids are ineffective in many patients with FSGS, and alternative treatments often yield suboptimal responses. Repository corticotropin injection (RCI; Acthar® Gel), a naturally sourced complex mixture of purified adrenocorticotropic hormone analogs and other pituitary peptides, may have beneficial effects on idiopathic FSGS via melanocortin receptor activation.

**Methods:**

Two studies in a preclinical (female Sprague-Dawley rats) puromycin aminonucleoside FSGS model assessed the effect of RCI on renal function and morphology: an 8-week comparison of a single RCI dose with methylprednisolone (*N* = 27), and a 12-week chronic RCI dose range study (*N* = 34). Primary outcomes were proteinuria and renal pathology improvements for measures of renal fibrosis, tubular damage, glomerular injury, and total kidney injury score. Impact of RCI treatment was also determined by assessing urinary biomarkers for renal injury, podocyte expression of podoplanin (a biomarker for injury), podocyte effacement by electron microscopy, and histological staining for fibrosis biomarkers.

**Results:**

Compared with saline treatment, RCI 30 IU/kg significantly reduced proteinuria, with a 38% reduction in peak mean urine protein levels on day 28 in the 8-week model, and RCI 10 IU/kg, 30 IU/kg, and 60 IU/kg reduced peak mean urine protein in the 12-week model by 18, 47, and 44%, respectively. RCI also showed significant dose-dependent improvements in fibrosis, interstitial inflammation, tubular injury, and glomerular changes. Total kidney injury score (calculated from histopathological evaluations) demonstrated statistically significant improvements with RCI 30 IU/kg in the 8-week study and RCI 60 IU/kg in the 12-week study. RCI treatment improved levels of urinary biomarkers of kidney injury (KIM-1 and OPN), expression of podoplanin, and podocyte morphology. RCI also reduced levels of desmin and fibrosis-associated collagen deposition staining. Methylprednisolone did not improve renal function or pathology in this model.

**Conclusions:**

These results provide evidence supporting the improvement of FSGS with RCI, which was superior to corticosteroid treatment in this experimental model. To the authors’ knowledge, this is the first evidence that a drug for the treatment of FSGS supports podocyte recovery after repeated injury.

## Background

### Focal segmental glomerulosclerosis

Focal segmental glomerulosclerosis (FSGS) is a disease that attacks kidney glomeruli and causes scarring, leading to kidney damage. FSGS is a renal condition frequently associated with nephrotic syndrome (NS) and is one of the most common forms of glomerular disease [1, 2]. FSGS has been observed in up to 35% of biopsies obtained from adult patients with idiopathic NS, and recent epidemiological data suggest the prevalence of FSGS is increasing [3, 4]. Histologically, FSGS is characterized by focal lesions of the glomeruli due to podocyte damage, which correlates with loss of function in glomerular permeability, proteinuria, and cell death [1–3]. FSGS and its most common complication, proteinuria, can be difficult to treat. FSGS shows the largest decline in estimated glomerular filtration rate and highest rate of progression to end-stage renal disease among glomerulopathies [5].

### Current FSGS treatment

First-line treatment for idiopathic FSGS is oral corticosteroids [6]. The mechanism of these drugs is unclear, but they are believed to act through potent immunosuppression and decreased nuclear factor–kappa B (NF-κB)–induced cytokine production [7]. Unfortunately, many patients develop steroid-resistant or steroid-dependent FSGS. Responses to steroids are lower in adult patients with FSGS versus other NSs, with a relapse rate of 47% in initial responders and 40–60% showing no improvement after 4 months [8]. The alternatives for patients with steroid-resistant FSGS include stronger immunosuppressants, such as calcineurin inhibitors, alkylating agents, and mycophenolate mofetil. However, these are associated with adverse effects and variable efficacy, which highlights the need for additional safe and effective options [6, 8]. Recently, an expert panel of nephrologists provided guidance on treatment of patients with NS [9]. The authors emphasized selecting therapies on the basis of disease pathophysiology and highlighted promising treatments not included in the current Kidney Disease Improving Global Outcomes guidelines, such as repository corticotropin injection (RCI; Acthar® Gel, Mallinckrodt Pharmaceuticals, Bedminster, NJ) [10].

### Repository corticotropin injection

RCI contains a naturally sourced complex mixture of purified adrenocorticotropic hormone analogues and other pituitary peptides [11]. RCI is indicated to induce remission of proteinuria in either NS without uremia of the idiopathic type or NS due to lupus erythematosus [11]. An increasing body of evidence has demonstrated the efficacy of RCI in difficult-to-treat patients with steroid-resistant or steroid-dependent glomerulopathies who failed standard first-line treatments. Tumlin et al. (2017) showed a significant overall reduction in proteinuria (*p* < 0.001), with 62% partial response and 8% complete response at 6 months with RCI in 13 FSGS patients who had failed multiple first-line therapies [12]. Similar results were seen in 9 patients with treatment-resistant idiopathic membranous nephropathy (iMN), with decreased proteinuria (*p* = 0.013) and a 44% partial response, which improved further with the addition of tacrolimus [12]. This supports previous findings wherein 9 of 11 patients with iMN and 1 of 1 patient with FSGS who failed an average of 2–3 immunosuppressive therapies achieved partial or complete remission (55 and 27%, respectively) [13, 14].

RCI activates all 5 melanocortin receptors (MCRs), each with a unique function. MC2R is the main target of activation in the hypothalamus-pituitary-adrenal axis leading to adrenal steroidogenesis and release [15, 16]. Autonomic and neuroendocrine functions appear to be heavily regulated by MC4R [17], whereas MC1R, MC3R, and MC5R are involved in steroid-independent immunomodulation of immune cells [18]. Increasing evidence suggests that MC1R activation alone may serve as a podocyte-specific target in kidney injury [19–22].

The efficacy of MCR agonists was originally thought to be due to their steroidogenic activity through MC2R. However, Lal and colleagues [23] showed an almost 37 times greater total steroid exposure with methylprednisolone (MP) treatment compared with RCI treatment, with clinically relevant doses for multiple sclerosis (MS). In light of this finding and owing to the functional differences between endogenous cortisol activation and exogenous corticosteroids, the mechanism of action of RCI extends beyond steroidogenesis. Clinical and experimental evidence continues to suggest a potential use for RCI in treating NS and idiopathic FSGS, particularly in patients with disease resistant to steroids and other immunosuppressants [13, 24–26]. Previous studies have demonstrated that RCI inhibits NF-κB activity and suppresses proinflammatory cytokine production from immune cells, which suggests that RCI might be superior to corticosteroids for the treatment of renal fibrosis [27]. However, evidence from both preclinical and clinical studies is limited, and the exact mechanisms through which RCI demonstrates efficacy in patients with steroid resistance are still unclear.

### Central hypothesis

Our hypothesis was that RCI, as a pan-MCR receptor agonist, would be superior to corticosteroids on podocyte function, renal fibrosis, and proteinuria in an experimental animal model containing podocyte damage and FSGS-type lesions characteristic of NS.

## Methods

### Preclinical PAN model

**Fig. 1:**
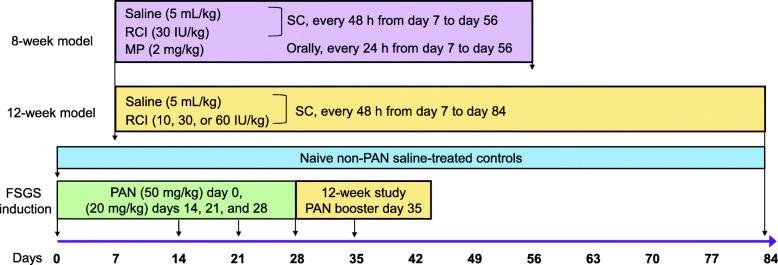
Study Design for the 8-Week and 12-Week Rat PAN FSGS Models. Abbreviations: FSGS, focal segmental glomerulosclerosis; MP, methylprednisolone; PAN, puromycin aminonucleoside; RCI, repository corticotropin injection; SC, subcutaneous

The current study used a modified rat puromycin aminonucleoside (PAN) model, resulting in FSGS-type lesions and podocyte damage observed in NS [1, 28, 29]. Disease induction for rodent FSGS has been described previously, with modification to dosage of PAN and route of delivery. We selected intravenous (IV) PAN delivery at 50 mg/kg with booster doses of 20 mg/kg to induce FSGS instead of one-time dosing that results in a minimal change disease phenotype [28, 30]. Two studies were conducted with female Sprague-Dawley rats (Envigo RMS, LLC, Indianapolis, Indiana, USA; age: 6–8 weeks; weight: 166–232 g). Due to the schedule of ongoing studies, the rats used in the 12-week study were slightly younger than those in the 8-week study. All animals were delivered with documentation confirming that they were treatment-naive and free of all tested pathogens. The first study used an 8-week PAN model, which primarily compared RCI with MP (*N* = 27; *n* = 3 naive, *n* = 8 each for saline [diseased], RCI 30 IU/kg, and MP 2 mg/kg). The second study used a long-term 12-week PAN model (*N* = 34; *n* = 4 naive, *n* = 6 for RCI 30 IU/kg, *n* = 8 each for saline, RCI 10 IU/kg, and 60 IU/kg) with an extended dose range of RCI for the treatment of FSGS. Disease induction with PAN occurred on day 0 (50 mg/kg), with treatment starting on day 7. Reinjury occurred with lower-dose PAN (20 mg/kg) on days 14, 21, and 28 in the 8-week model, and additional injury occurred on day 35 in the 12-week model (Fig. 1). Naive controls did not receive PAN induction. Animals were sacrificed in a controlled delivery system using isoflurane followed by exsanguination.

During the 8- and 12-week studies, multiple disease endpoints were analyzed to assess renal function and kidney morphology. Primary dependent measures were levels of proteinuria and scores for renal fibrosis, tubular damage, and glomerular injury. A score for interstitial inflammation was also measured, and the 4 scores were summed to yield a total kidney injury score. Scoring was performed by a certified pathologist and was graded from 0 (normal) to 5 (severe).

### Experimental conditions and locations

All experiments were performed between 8 am and 5 pm central standard time. Rats were housed 3 or 4 animals per cage in standard shoebox cages (16x8x8 inches). Aspen shavings were used for bedding in home cages. All study animals were kept on a diurnal 12-h light/dark cycle, with ample light used for routine inspection and cleaning. Ambient temperatures were maintained between 65 °F and 85 °F. Standard rat chow pellets were given freely. All animals had ad libitum access to potable water via water bottle. Rats were provided with enrichment devices such as Nylabone® products, paper twists, or other devices approved by the attending vet.

All in-life experiments were conducted in the site vivarium, with dosing and urine collection completed in the controlled procedure room. Animals were observed daily, including weekends and holidays, to assess general health and well-being and were weighed at least twice per week.

All rat urine assays were performed with 24-h urine collections in metabolism cages with 1 rat per cage. Animals were normalized across 24 h in the metabolism cages, urine tubes were replaced, and animals remained in the metabolism cages for an additional 24 h for the duration of the urine collection. Animals had unlimited access to food and water. All histology procedures, including immunohistochemistry (IHC) staining, were completed on site. Enzyme-linked immunosorbent assay (ELISA) was conducted in the laboratory space adjoining the Meso Scale Discovery platform. All image analyses were completed in a designated imaging room on-site. All experiments were completed in the same building, on the same floor, and in the same wing of the floor.

### Sample size determination

Animal sample size was determined using the resource equation method, with consideration of minimizing unnecessary animal usage, per our site guidelines [31]. This equation uses the following formula: E (degree of freedom of analysis of variance) = TA (total animals) – TG (total number of groups). For the 8-week study, 27 (TA) – 4 (TG) = 23. For the 12-week study, 34 (TA) – 5 (TG) = 29. Values greater than 20 are considered good. As naive samples were only included to confirm baseline non-disease parameters, limited numbers were used in accordance with ethical guidance to limit unnecessary animal usage.

### Study drugs and treatment regimens

Dosing methodology was selected on the basis of normal treatment routes of administration. For example, in clinical settings, RCI is given subcutaneously. The most common route of administration of corticosteroids is oral dosing. The doses for RCI were determined by multiple previous in-house studies across animal models that have shown efficacy. As for MP dosing, high-dose treatment in human FSGS for prednisone is 1 mg/kg/day, with lower doses of 0.5 mg/kg/day used for maintenance [32]. Rat dosing translates to approximately 3 to 6 mg/kg/day of prednisone, or ~ 2 to 5 mg/kg/day of MP. We therefore selected 2 mg/kg/day of MP to avoid side effects of high-dose steroid treatment and to use a dose that is in line with other animal models of kidney injury.

RCI (10, 30, and 60 IU/kg) and saline were administered subcutaneously, whereas MP was administered by oral gavage. In the 8-week model, FSGS was induced with 4 IV doses of PAN (days 0, 14, 21, and 28). RCI (30 IU/kg) was administered every other day beginning on day 7, whereas MP 2 mg/kg was given daily beginning on day 7. In the 12-week model, FSGS was induced with 5 IV doses of PAN (days 0, 14, 21, 28, and 35), and RCI (10, 30, and 60 IU/kg) was administered every other day beginning on day 7. Naive rats were treated with saline and no PAN to mimic handling of experimental rats (Fig. 1).

All animals were treated in the morning. On days when both RCI and MP treatments were given, animals were treated by group at the same time. Animals were assessed daily by visual inspection.

### Urine protein collection and creatinine assays

Urine was collected biweekly for the 8-week PAN study and weekly for the 12-week PAN study. On urine collection days, urine was initially discarded while animals acclimated to metabolic cages for 24 h. Over the next 24 h, urine was collected, centrifuged, aliquoted, and stored at − 80 °C until processing. Total urine protein was measured using the pyrogallol red-molybdate dye-binding method (BioAssay Systems, Hayward, CA, Cat. #QTPR001), and 24-h total protein was calculated to assess proteinuria. Urine creatinine (uCr) was determined with the Jaffe method, a colorimetric plate-based assay (BioAssay Systems, Hayward, CA, Cat. #DICT015). Two technical replicates were included for each sample, with the average of the replicates used for the final value.

### Meso Scale multiplex ELISA

Urinalysis of kidney injury biomarkers osteopontin (OPN) and kidney injury molecule-1 (KIM-1) was performed via multiplex ELISA on the Meso Scale Diagnostics (Rockville, MD) Discovery system. KIM-1 and OPN were evaluated on day 28 after the third PAN booster dose (20 mg/kg), at peak proteinuria in the 8-week model, and again at day 56 after 4 weeks of treatment following the final PAN injection. An additional time point at day 42 was added for the 12-week model, corresponding to peak proteinuria in the study. Samples were tested via rat Kidney Injury Panel-1 (Cat. #K15162C, Meso Scale Diagnostics, Rockville, MD), and values were expressed as ng/mL. The calculated values from the ELISA were normalized for each animal to the uCr concentrations in mg/mL, resulting in a ratio of ng:mg of target:creatinine concentration. Two technical replicates were included for each sample, with the average of the replicates used for the final value. Because the efficacy comparison was only in the PAN treatment (diseased) groups, naive (non-diseased; non-treated) samples were pooled between the 8- and 12-week studies for ELISA comparisons to gain a more robust negative control baseline. Little variation in naive samples was expected based on previous experience of standard values for this age range, and animal numbers were limited in each study for ethical reasons.

### Histology, pathology, and IHC procedures

Animals were sacrificed, and kidneys were immediately removed. Cross sections of the central portion of the left and right kidneys were fixed in 10% neutral buffered formalin and processed to paraffin blocks for routine histology and IHC. In the 12-week PAN study, additional portions of renal cortex from both the left and right kidneys were fixed for analysis by electron microscopy (EM). All kidneys were stained with haematoxylin and eosin (H&E) and periodic acid-Schiff (PAS) for pathology scoring; Masson’s trichrome, Sirius Red/Fast-Green, and IHC staining were used to assess fibrosis and podocyte biomarkers. Slides stained with H&E, PAS, and Masson’s trichrome were sent to Seventh Wave Laboratories (Maryland Heights, MO) for a renal pathology assessment by a board-certified veterinary pathologist who was not blinded to treatment groups, but did not have details on the specific study drug. EM samples for the 12-week study were sent to Charles River Laboratories (Durham, NC), where they were evaluated for treatment-related changes to the glomerulus by a board-certified veterinary pathologist who was not blinded to treatment groups. All IHC staining (eg, for podoplanin [PDPN]) was performed on the Ventana (Oro Valley, AZ) Discovery Ultra automated stainer. The antibody used for PDPN was Novus (Centennial, CO) NB110–962423.

### Quantitative image analysis

Slides were scanned on a NanoZoomer S210 (Hamamatsu Photonics, Hamamatsu, Japan) at 20x or 40x magnification. Tissue images were analyzed through Visiopharm (Hørsholm, Denmark) quantitative image analysis software. For quantification of fibrosis, the kidney cortex was outlined at the region of interest (ROI), and interstitial fibrosis was quantitated per area of total tissue. Glomeruli from all areas of the kidney cortex were selected as ROIs from both the left and right kidney sections. An average of ~ 200 glomeruli per animal was selected to decrease bias due to focal pathology. Two individuals randomly identified the ROIs used in the final analyses. The software was trained to identify and quantitate staining of interest. All slides were batch processed using the same algorithm for all slides in each analysis. Naive samples were pooled for imaging analysis across studies, similar to the ELISA analysis.

### Statistical analysis

Comparisons between groups were performed through 1-way or 2-way analysis of variance (ANOVA) and to check the assumption of equal variances, with appropriate post hoc tests for multiple comparisons or *t* tests as indicated for continuous normally distributed data. Non-normally distributed continuous and categorical data were compared with nonparametric Kruskal-Wallis (ANOVA) with post hoc tests, Mann-Whitney for 2-group comparison, or Fisher’s exact test. Graphs are presented as mean ± standard error of the mean unless otherwise noted. A significance level of *p* < 0.05 was established for all statistical tests.

### Protocol deviations

In the 8-week study, one naive control died after routine bleeding, which was categorized as a laboratory handling error. In the 12-week study, 2 animals in the RCI 30 IU/kg group lost more than 20% of their body weight by day 47 or day 49 and were sacrificed according to our humane animal use protocol. Both animals had elevated proteinuria from disease induction, even prior to treatment, and maintained elevated proteinuria levels compared with all groups until their death. The lack of any animal deaths in the higher dose group of 60 IU/kg suggests the deaths were not likely treatment related but were caused by an extreme sensitivity to PAN injury. These animals were not included in any analyses. No additional controls or modifications were needed in this study, as the animal use guidelines implemented here account for changes in animal health, and the number of unexpected deaths was low (3/64 animals).

### Ethical considerations

All study procedures, including for animal use welfare, were performed in accordance with the National Institutes of Health’s Guide for the Care and Use of Laboratory Animals. The study protocol was reviewed and approved by the Institutional Animal Care and Use Committee of Mallinckrodt Pharmaceuticals.

## Results

### Baseline characteristics

Animals were randomized by urine protein levels to ensure no significant differences were observed between groups. Baseline body weights and urine protein were measured before disease induction (Table 1).

**Table 1:** Baseline characteristics of the 8-week and 12-week PAN models

Parameter	8-week PAN model				12-week PAN model				
	Mean ± SD								
	Naive	Saline	RCI 30 IU/kg/qod	MP 2 mg/kg/qd	Naive	Saline	RCI 10 IU/kg/qod	RCI 30 IU/kg/qod	RCI 60 IU/kg/qod
n	3	8	8	8	4	8	8	6	8
Body Weight (g)	220.2 ± 11.6	208.0 ± 7.0	207.0 ± 10.6	212.9 ± 7.0	177.1 ± 7.1	181.1 ± 5.7	178.7 ± 7.8	176.7 ± 4.6	176.9 ± 6.4
Urine Protein (mg/24 h)	2.8 ± 1.1	3.2 ± 1.5	2.9 ± 0.6	3.3 ± 1.2	1.7 ± 0.6	1.1 ± 0.4	1.0 ± 0.5	1.3 ± 1.0	1.1 ± 0.3

No significant differences were observed between groups for mean weight or baseline urine protein

*Abbreviations*: *MP* Methylprednisolone, *PAN* Puromycin aminonucleoside, *RCI* Repository corticotropin injection, *SD* Standard deviation, *qod* Every other day, *qd* Per day

### RCI effect on renal function in PAN-FSGS models

**Fig. 2:**
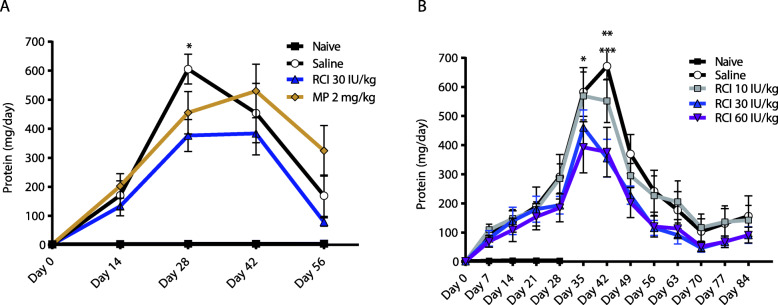
Proteinuria in the 8-Week and 12-Week PAN Models. **a** Protein concentration (mg/24 h) in urine over the 8-week PAN study (*p < 0.05 day 28 for RCI 30 IU/kg versus saline; 2-way ANOVA, Bonferroni’s post hoc comparison). **b** Protein concentration (mg/24 h) in urine over the 12-week study (*p < 0.05 day 35 for 60 IU/kg RCI; **p < 0.001 day 42 for 30 IU/kg RCI; ***p < 0.0001 day 42 for RCI 60 IU/kg; all versus saline; 2-way ANOVA, Bonferroni’s post hoc comparisons). In both panels, values are mean ± standard error of the mean. Abbreviations: ANOVA, analysis of variance; MP, methylprednisolone; PAN, puromycin aminonucleoside; RCI, repository corticotropin injection

Naive (non-diseased) rats were assessed for comparison throughout our studies to confirm the disease phenotype induced by PAN. Treatment of rats with puromycin at 50 mg/kg at day 0, followed by booster doses of 20 mg/kg at days 14, 21, and 28 in the 8-week model and an additional dose at day 35 in the 12-week model, resulted in maximum urine protein levels of approximately 600–700 mg/24 h. RCI treatment of 30 IU/kg significantly reduced proteinuria (*p* < 0.05), with a 38% reduction in peak mean urine protein levels at day 28 compared with saline and a 29% total reduction in area under the curve (AUC) over the study period (12,843 mg/24 h*d vs 18,107 mg/24 h*d) in the 8-week model (Fig. 2a). We also observed an initial 25% decrease in peak urine protein levels with MP treatment of 2 mg/kg/d at day 28, but this reduction was lost by day 42, and levels were nearly 2-fold greater than with saline at day 56, with similar total AUC as saline over the study duration (18,486 mg/24 h*d MP treatment vs 18,107 mg/24 h*d saline treatment). MP treatment showed nearly 4-fold higher urine protein levels than with RCI at day 56. In the 12-week RCI multidose model, peak urine protein at day 42 was reduced 18% versus saline at the lowest dose tested of 10 IU/kg, with significant reductions of 47 and 44% from peak levels at the 30 IU/kg and 60 IU/kg dose levels, respectively (*p* < 0.001 for each, Fig. 2b). Proteinuria AUC for the 12-week study was reduced by 35 and 39% in the 30 IU/kg and 60 IU/kg groups, respectively, compared with saline (13,769 mg/24 h*d and 12,987 mg/24 h*d versus 21,139 mg/24 h*d).

### Effects of RCI on kidney injury in PAN-FSGS models

In PAN-treated rats, mild to moderate increases in renal fibrosis, tubular injury, and glomerular changes were observed by histopathologic analysis, with a minimal to mild increase in interstitial inflammation in both the 8-week and 12-week models. RCI treatment at 30 or 60 IU/kg reduced these pathologic conditions, with dose-dependent decreases in the 12-week study (Table 2). The calculated total kidney injury score was significantly lower with RCI 30 IU/kg in the 8-week model versus saline, mean ± standard deviation of 5.9 ± 1.4 vs 8.8 ± 2.1 (*p* < 0.05), and with RCI 60 IU/kg in the 12-week model versus saline, 5.1 ± 2.5 vs 8.8 ± 2.4 (*p* < 0.05). Treatment with MP did not reduce disease histopathology or the calculated total kidney injury score compared with saline in the 8-week model (Table 2).

**Table 2:** Histopathological evaluation injury scores of H&E-stained kidney sections for the 8-week and 12-week PAN models

	Parameter	8-week PAN model			12-week PAN model			
		Mean ± SD						
		Saline	MP 2 mg/kg/qd	RCI 30 IU/kg/qod	Saline	RCI 10 IU/kg/qod	RCI 30 IU/kg/qod	RCI 60 IU/kg/qod
Pathology Report	Fibrosis	2.3 ± 0.5	2.9 ± 0.8	^a^1.6 ± 0.5	2.3 ± 0.7	2.1 ± 1.0	^a^1.7 ± 0.5	^a^1.3 ± 0.7
	Interstitial Inflammation	1.4 ± 0.5	1.3 ± 0.5	1.0 ± 0.0	1.4 ± 0.5	1.1 ± 0.3	1.0 ± 0.0	^a^0.6 ± 0.7
	Tubular Injury	2.6 ± 0.5	2.8 ± 0.9	^a^1.5 ± 0.5	2.4 ± 0.5	2.4 ± 0.7	^a^1.7 ± 0.5	^a^1.3 ± 0.5
	Glomerular Changes	2.5 ± 0.9	3.3 ± 0.7	^a^1.8 ± 0.5	2.8 ± 1.0	3.0 ± 0.9	^a^2.3 ± 0.5	^a^2.0 ± 0.9
Quantitative	Total Kidney Injury Score	8.8 ± 2.1	10.1 ± 2.7	*5.9 ± 1.4	8.8 ± 2.4	8.6 ± 2.9	6.7 ± 1.4	*5.1 ± 2.5

Total kidney injury score was calculated as the mean of all summed severity scores per animal, derived from the official pathology report. Severity scores ranged from 0 to 5 (0 = Normal, 1 = Minimal, 2 = Mild, 3 = Moderate, 4 = Marked, 5 = Severe)

*Abbreviations*: *ANOVA* Analysis of variance, *H&E* Haematoxylin and eosin, *MP* Methylprednisolone, *PAN* Puromycin aminonucleoside, *RCI* Repository corticotropin injection, *SD* Standard deviation, *qod* Every other day, *qd* Per day

*p < 0.05, Kruskal-Wallis nonparametric ANOVA, Dunn’s post hoc test, comparing the RCI treatment groups with saline

^a^Assessments that were considered significant by the pathologists in the official pathology report as a treatment-related difference from saline controls

**Fig. 3:**
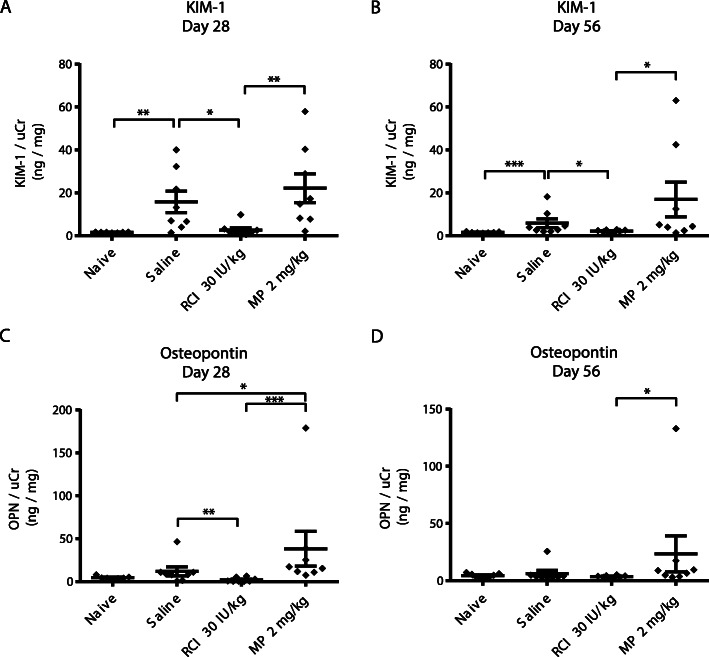
Quantification of Urine OPN and KIM-1 Levels in the 8-week PAN Model. **a**, **b** KIM-1 levels normalized to creatinine. **c**, **d** OPN levels normalized to creatinine. For all panels, the Mann-Whitney test was used to determine statistical significance, and all values are mean ± standard error of the mean. *p < 0.05; **p < 0.01; ***p < 0.001. Abbreviations: KIM-1, kidney injury molecule-1; MP, methylprednisolone; OPN, osteopontin; PAN, puromycin aminonucleoside; RCI, repository corticotropin injection; uCr, urine creatinine

**Fig. 4:**
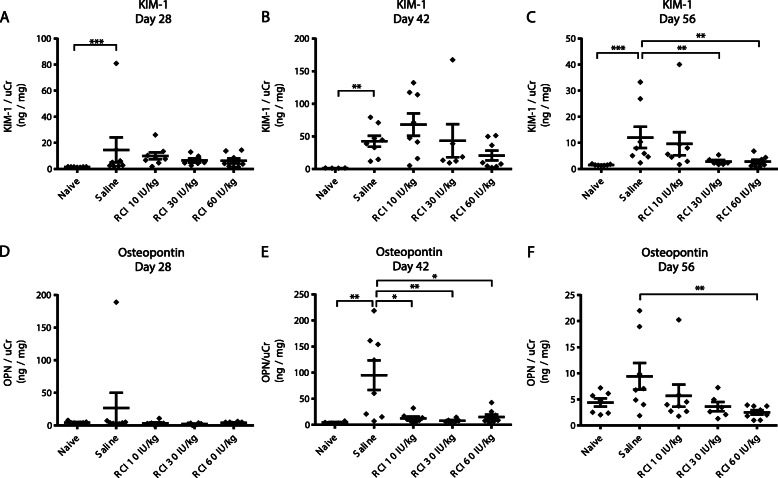
Quantification of Urine OPN and KIM-1 Levels in the 12-week PAN Model. **a**-**c** KIM-1 levels normalized to creatinine. **d**-**f** OPN levels normalized to creatinine. For all comparisons, the Mann-Whitney test was used to determine statistical significance. * *p* < 0.05; **p < 0.01; ***p < 0.001. In all panels, values are mean ± standard error of the mean. Abbreviations: KIM-1, kidney injury molecule-1; OPN, osteopontin; PAN, puromycin aminonucleoside; RCI, repository corticotropin injection; uCr, urine creatinine

RCI treatment also lowered urine biomarkers of kidney injury. Concentrations of KIM-1 and OPN normalized to uCr (KIM-1/uCr, ng/mg) were analyzed in both 8- and 12-week models (Figs. 3 and 4). Naive samples showed little variability for KIM-1 and OPN analyses (Figs. 3 and 4). KIM-1/uCr was significantly elevated after PAN treatment plus saline at all time points in both models compared with naive controls (*p* < 0.01) (Figs. 3a, b and 4a-c). Rats treated with RCI at 30 IU/kg had significantly lower KIM-1 concentrations compared with saline on days 28 and 56 in the 8-week study (*p* < 0.05), whereas MP treatment showed higher KIM-1 levels compared with saline on day 28 and 56 on average (Fig. 3a, b). Treatment with RCI doses of 30 and 60 IU/kg significantly reduced KIM-1 concentration compared with saline on day 56 in the 12-week model (*p* < 0.01) (Fig. 4c).

The kidney injury biomarker OPN was not significantly elevated after PAN treatment plus saline at day 28 or 56 in either model (Figs. 3c, d and 4d, f), but it was significantly higher at day 42 in the 12-week model (*p* < 0.01) (Fig. 4e). RCI 30 IU/kg significantly reduced OPN levels (*p* < 0.01) at day 28 in the 8-week model, whereas MP significantly increased OPN levels compared with saline (*p* < 0.05). Treatment with RCI significantly lowered OPN concentrations at day 42 for RCI 10, 30, and 60 IU/kg (*p* < 0.05) (Fig. 4e) and day 56 for RCI 60 IU/kg (*p* < 0.01) (Fig. 4f) compared with saline in the 12-week model.

**Fig. 5:**
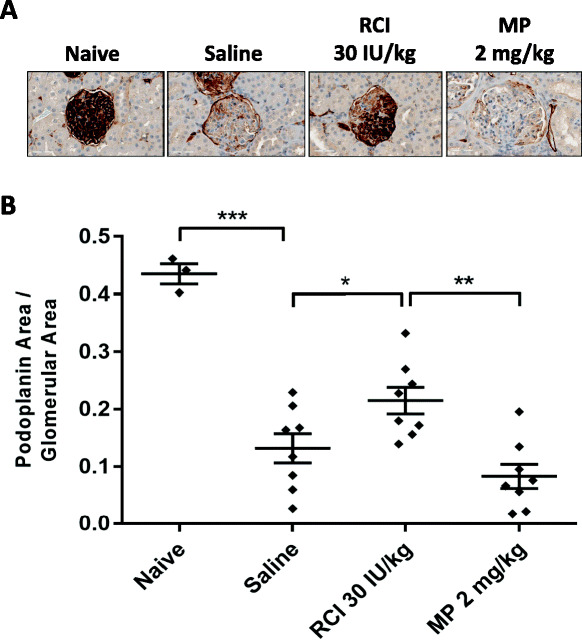
Podoplanin Expression in the 8-Week PAN Model. **a** Podoplanin expression via IHC staining. **b** IHC analysis of podoplanin levels presented as proportion of glomerular staining in kidney sections at day 56 (*p < 0.05; **p < 0.001; ***p < 0.0001). For all comparisons, unpaired t tests were used to determine statistical significance. Values are mean ± standard error of the mean. Abbreviations: IHC, immunohistochemistry; MP, methylprednisolone; PAN, puromycin aminonucleoside; RCI, repository corticotropin injection

**Fig. 6:**
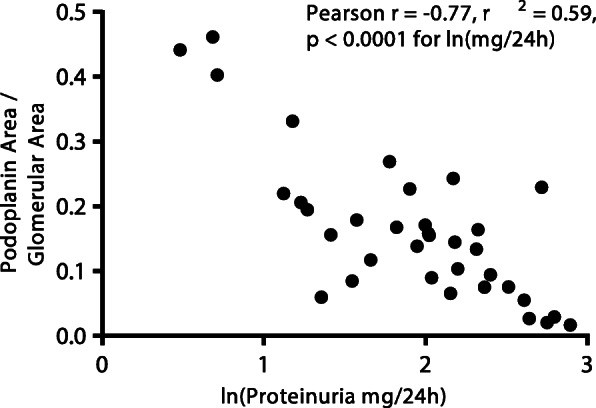
Urine Protein Concentration and Podoplanin Staining per Glomerular Area Correlation in the 8-Week PAN Model. There is a significant negative correlation between glomerular podoplanin staining, as a proportion of total glomerular area, and log-transformed 24-h urine protein. The relationship between the two variables was quantified using the Pearson correlation coefficient and statistical significance was determined by *t* test. Abbreviations: ln, natural log; PAN, puromycin aminonucleoside

In the 8-week model, RCI treatment protected against loss of PDPN expression, an indication of podocyte injury. PAN-induced renal injury decreased mean PDPN glomerular area staining by approximately 70% compared with naive (Fig. 5a, b). RCI 30 IU/kg treatment significantly increased PDPN glomerular expression compared with saline (*p* < 0.05), whereas MP treatment was unable to prevent PDPN loss, with lower mean area staining than saline (Fig. 5a, b). As a proportion of total glomerular area, glomerular PDPN staining was significantly negatively correlated to log-transformed 24-h urine protein (Pearson’s *r* = − 0.77, *p* < 0.0001) (Fig. 6). PDPN staining was evaluated in the 12-week model as well, but after the last PAN injection, PAN-injured animals showed normal expression of most podocyte markers comparable with naive controls after 7 weeks of recovery (data not shown).

### Effects of RCI on podocyte morphology in PAN-FSGS models

**Fig. 7:**
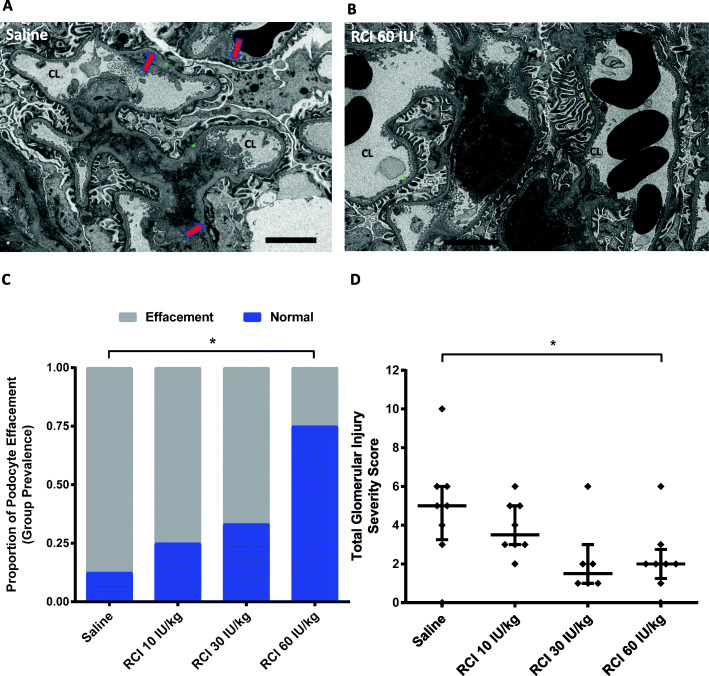
Podocyte and Glomerular Assessment in the 12-week PAN Model. **a** Saline EM image. Red arrows = podocyte effacement. **b** RCI 60 IU/kg EM image. Blue arrows = normal podocyte foot process structure. **a**, **b** Green * = capillary basement membrane; CL = capillary lumen; scale bar = 4 μM. **c** Group prevalence of podocyte effacement by EM analysis. *p < 0.05, Fisher’s exact test for group differences compared with saline, 2-tailed. **d** Total glomerular injury score. *p < 0.05, Kruskal-Wallis nonparametric ANOVA, Dunn’s post hoc test, comparing the RCI treatment groups with saline. Values are mean ± standard error of the mean. For all panels, naive samples are not shown because of low sample size. Abbreviations: ANOVA, analysis of variance; EM, electron microscopy; PAN, puromycin aminonucleoside; RCI, repository corticotropin injection

RCI treatment led to long-term benefits in podocyte morphology as demonstrated by EM analysis in the 12-week model. RCI treatment significantly decreased the prevalence of podocyte effacement (Fig. 7a-c) and total glomerular injury score in the 60 IU/kg dose group (*p* < 0.05) compared to saline (Fig. 7d).

### Effects of RCI on fibrosis markers in PAN-FSGS models

**Fig. 8:**
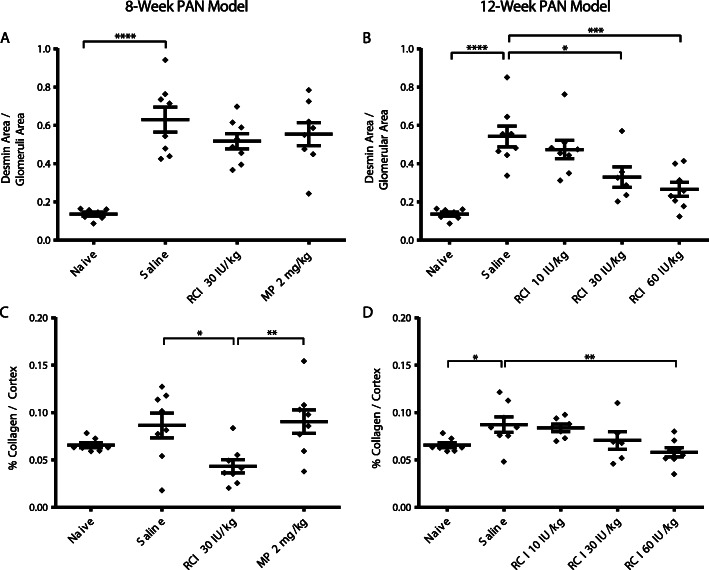
Fibrosis Marker Levels in the 8-week and 12-week PAN Models. **a**, **b** Image analysis of glomerular staining for desmin as a marker of epithelial-mesenchymal transition. **c**, **d** Sirius Red/Fast Green (collagen) deposition staining as a marker of fibrosis. For all comparisons, unpaired *t* tests were used to determine statistical significance. *p < 0.05; **p < 0.01; ***p < 0.001; ****p < 0.0001. Values are mean ± standard error of the mean. Abbreviations: MP, methylprednisolone; PAN, puromycin aminonucleoside; RCI, repository corticotropin injection

Quantitative image analysis of glomerular staining for epithelial-mesenchymal transition (EMT), a cellular mechanism of fibrosis [33], was also assessed in the kidney cortex in both models. Glomerular IHC staining of the EMT marker desmin was significantly elevated in PAN-treated animals (*p* < 0.0001) in both models (Fig. 8a, b). In the 12-week model, glomerular desmin staining was significantly lower for RCI 30 IU/kg (*p* < 0.05) and 60 IU/kg (*p* < 0.001) compared with saline (Fig. 8b). Sirius Red/Fast Green staining for collagen deposition, a marker for fibrosis, was significantly decreased with RCI 30 IU/kg versus saline (*p* < 0.05) in the 8-week model (Fig. 8c) and for RCI 60 IU/kg versus saline (*p* < 0.01) in the 12-week model (Fig. 8d).

## Discussion

Overall, RCI improved glomerular function and pathology after repeated PAN injury in Sprague-Dawley rats. RCI reduced proteinuria, improved EM measures of podocyte morphology, decreased urinary biomarkers of renal damage, and increased IHC markers of podocyte function. RCI also reduced histopathology scores of fibrosis, interstitial inflammation, tubular injury, and glomerular changes after injury. By comparison, MP treatment alone was not beneficial for any of the relevant endpoints in these models. The early benefit in proteinuria reduction by MP could not be maintained throughout the study, and overall renal injury in this group was no better than in saline controls, often with higher mean severity scores than no treatment.

Importantly, the improvements observed with RCI occur *after* podocyte damage in a rodent model of FSGS. Rather than simply preventing symptoms of FSGS, RCI was shown to reverse damage to podocytes, with improved podocyte structure and function even after repeated renal injury in the 12-week model. These preclinical models demonstrate the therapeutic benefit of RCI in treatment of ongoing FSGS. The authors are not aware of another FSGS drug shown to improve damage to podocytes after repeated injury.

Results from this study are similar to published data showing statistically significant reductions in proteinuria with RCI in patients with FSGS [34, 35]. In addition, decreases in levels of KIM-1 (a tubular injury marker) and OPN (a glycoprotein associated with podocyte damage) with RCI treatment in this study are consistent with the previous characterization of KIM-1 and OPN as biomarkers of glomerular disease [36–38] and of acute kidney injury [39]. Although the increase in KIM-1 with MP compared with saline on days 28 and 56 was unexpected, the variance for KIM-1 in the MP groups was generally high, and the literature suggests that KIM-1 may have an unpredictable response to glucocorticoids. A patient with tubulointerstitial nephritis and uveitis showed fluctuations in KIM-1 after glucocorticoid treatment [40].

These results indicate that in an extended non–immune-mediated model of FSGS, steroid-mediated adverse effects from chronic treatment may outweigh early efficacy seen in FSGS models of shorter duration [41]. Although MP did not show sustained effect on proteinuria in this model, a randomized trial in patients with membranous nephropathy showed similar efficacy for RCI and MP on proteinuria remission [42]. The discrepancy in study results could be explained by differences across species or in study protocol; patients receiving MP in the membranous nephropathy study alternated the drug with cyclophosphamide or chlorambucil.

The increased PDPN expression demonstrated in this study may be a benefit of MCR-mediated RhoA activation, explored in previous studies [15–18]. The literature [43] and this study suggest that melanocortin receptor activation can improve renal disease with or without endogenous steroid production, with observed direct podocyte effects beyond general immunosuppression. The improvements in proteinuria and glomerular morphology observed in this study could be mediated specifically by RCI-dependent activation of MC1R. In a rodent model of membranous nephropathy (passive Heymann nephritis), an MC1R agonist improved proteinuria and glomerular morphology [19]. However, in a rodent model of FSGS (adriamycin), MC1R agonists did not reduce albuminuria (a type of proteinuria) [20]. Differences in the animal models may account for these discrepancies. Further studies are warranted to identify specific RCI-dependent MCR activity in FSGS.

### Limitations

Although a steroid treatment condition was included in the 8-week PAN group to model traditional steroid treatment, the 12-week PAN model did not include an MP group for comparison and was designed as a dose-response study for RCI. The two studies also showed slight differences in baseline age and weight. Kidney function was evaluated by proteinuria; additional measures such as blood urea nitrogen, serum creatinine, and urinary albumin were not included.

## Conclusions

In the current study, RCI treatment was effective in reducing PAN-induced renal damage and podocyte injury, which were not observed by treatment with MP. These results provide further evidence for a unique mechanism of MCR agonism in the treatment of FSGS, which was superior to steroid treatment in this experimental model.

## Data Availability

The datasets used and/or analyzed during the current study are available from the corresponding author on reasonable request.

## Abbreviations

ANOVA: Analysis of variance
AUC: Area under the curve
ELISA: Enzyme-linked immunosorbent assay
EM: Electron microscopy
EMT: Epithelial-mesenchymal transition
FSGS: Focal segmental glomerulosclerosis
H&E: Haematoxylin and eosin
iMN: Idiopathic membranous nephropathy
IHC: Immunohistochemistry
IV: Intravenous
KIM-1: Kidney injury molecule-1
MCR: Melanocortin receptor
MP: Methylprednisolone
MS: Multiple sclerosis
NF-κB: Nuclear factor–kappa B
NS: Nephrotic syndrome
OPN: Osteopontin
PAN: Puromycin aminonucleoside
PAS: Periodic acid-Schiff
PDPN: Podoplanin
RCI: Repository corticotropin injection
ROI: Region of interest
uCr: Urine creatinine
