# Correction to: Repository corticotropin injection versus corticosteroids for protection against renal damage in a focal segmental glomerulosclerosis rodent model

**DOI:** 10.1186/s12882-020-01933-3

**Published:** 2020-07-16

**Authors:** Kyle Hayes, Elizabeth Warner, Chris Bollinger, Dale Wright, Richard M. Fitch

**Affiliations:** Mallinckrodt Pharmaceuticals, 675 James S. McDonnell Blvd, 20-1-W, Hazelwood, MO USA

**Correction to: BMC Nephrology (2020) 21:226**

**https://doi.org/10.1186/s12882-020-01879-6**

Following publication of the original article [1], the authors identified an error in Fig. 7. The correct figure is given below.

**Fig. 7:**
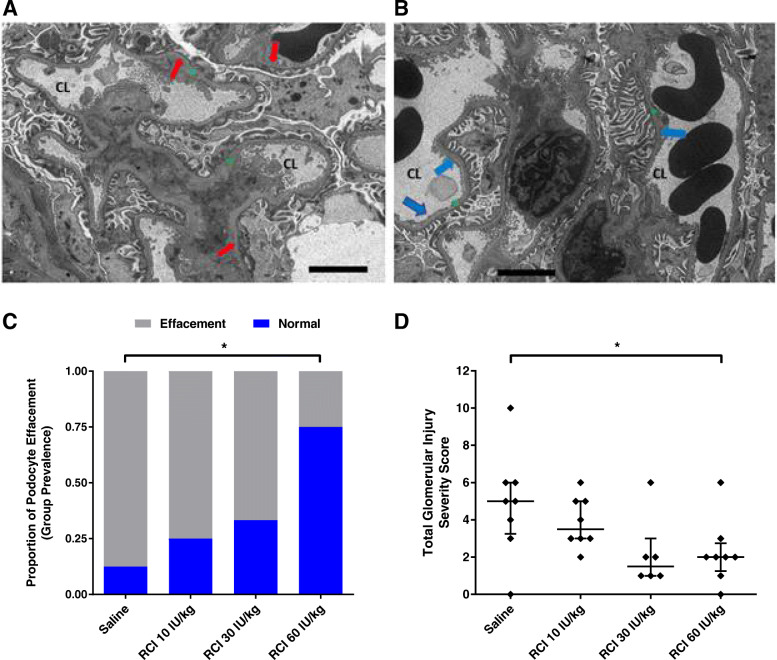
Podocyte and Glomerular Assessment in the 12-week PAN Model. **a** Saline EM image. Red arrows = podocyte effacement. **b** RCI 60 IU/kg EM image. Blue arrows = normal podocyte foot process structure. **a**, **b** Green * = capillary basement membrane; CL = capillary lumen; scale bar = 4 μM. **c** Group prevalence of podocyte effacement by EM analysis. *p < 0.05, Fisher’s exact test for group differences compared with saline, 2-tailed. **d** Total glomerular injury score. *p < 0.05, Kruskal-Wallis nonparametric ANOVA, Dunn’s post hoc test, comparing the RCI treatment groups with saline. Values are mean ± standard error of the mean. For all panels, naive samples are not shown because of low sample size. Abbreviations: ANOVA, analysis of variance; EM, electron microscopy; PAN, puromycin aminonucleoside; RCI, repository corticotropin injection

The original article has been corrected.
